# Numerical Calculation of the Irreversible Entropy Production of Additively Manufacturable Off-Set Strip Fin Heat-Transferring Structures

**DOI:** 10.3390/e25010162

**Published:** 2023-01-13

**Authors:** Marco Fuchs, Nico Lubos, Stephan Kabelac

**Affiliations:** Institute of Thermodynamics, Leibniz University Hannover, An der Universität 1, 30823 Garbsen, Germany

**Keywords:** off-set strip fin structures, numerical calculation, irreversible entropy production rate, shear stresses, heat conduction, additive manufacturing

## Abstract

In this manuscript, off-set strip fin structures are presented which are adapted to the possibilities of additive manufacturing. For this purpose, the geometric parameters, including fin height, fin spacing, fin length, and fin longitudinal displacement, are varied, and the Colburn j-factor and the Fanning friction factor are numerically calculated in the Reynolds number range of 80–920. The structures are classified with respect to their entropy production number according to Bejan. This method is compared with the results from partial differential equations for the calculation of the irreversible entropy production rate due to shear stresses and heat conduction. This study reveals that the chosen temperature difference leads to deviation in terms of entropy production due to heat conduction, whereas the dissipation by shear stresses shows only small deviations of less than 2%. It is further shown that the variation in fin height and fin spacing has only a small influence on heat transfer and pressure drop, while a variation in fin length and fin longitudinal displacement shows a larger influence. With respect to the entropy production number, short and long fins, as well as large fin spacing and fin longitudinal displacement, are shown to be beneficial. A detailed examination of a single structure shows that the entropy production rate due to heat conduction is dominated by the entropy production rate in the wall, while the fluid has only a minor influence.

## 1. Introduction

With the possibilities of the additive manufacturing technology, new heat-transferring structures can be developed, and existing structures can be optimized with regard to heat transfer and pressure drop [1,2]. At the same time, these possibilities free up many new geometric parameters that can be varied when optimizing heat transfer and pressure drop [3,4,5,6]. A suitable evaluation method is important on this behalf with respect to the effectiveness to check on the improvements made.

For this purpose, the calculation of the entropy production rate is suitable since this is influenced by both the heat transfer and the pressure drop. Therefore, optimization with respect to entropy production rate consistently leads to efficient heat transfer as well as low pressure drops [7].

The idea behind this evaluation has already been taken up by various authors. Bejan introduced the irreversible entropy production number (or entropy production number) [8], which puts the irreversible entropy production rate in relation to the transferred entropy flow. Furthermore, this approach allows the separation of the irreversible entropy production rate due to heat transfer and pressure drop to evaluate different structures and, in turn, to derive the optimal measures. Further developments of this kind have been proposed by Bejan and Pfister [9] and Yujie et al. [10], among others, in order to compare optimized geometries integrally with reference geometries.

Further investigations based on an integral 2nd law analysis were carried out by Aasi and Mishra [11] on heat exchangers with three-fluid flows, where different fin structures were investigated with respect to their entropy generation. Djetel-Gothe et al. [12] studied the irreversible entropy generation of a cooler in a Stirling engine by varying the parameters of mass flow, temperature, hydraulic diameter, and heat-transferring length. Zainith et al. [13] investigated the influence of different nanofluids in a spiral heat exchanger on exergy losses, and Rashidi et al. [14] performed an exergy analysis on a shell-and-tube heat exchanger. El Jery et al. [15] performed a study on the entropy generation of Al_2_O_3_-water nanofluid in internal flows with different shapes. Moreover, the principle of irreversible entropy production rate can be transported to a local level, making it possible to find the exact causes and positions of irreversible entropy production rate [16]. An extension of this principle to Reynolds-averaged flows was made by Kock et al. [17,18,19]. Thus, a separation can be made regardless of whether the entropy production rate occurs as a result of temperature gradients, i.e., heat conduction, or of shear stresses, i.e., as friction; furthermore, it can be determined whether the entropy production rate is caused by turbulence-induced fluctuations or molecular effects [20,21,22]. This principle is taken up by Ji et al. [23] to determine what causes an entropy production rate to occur in flows with nanofluids and how the entropy production rate develops in the boundary layer. Furthermore, Ji et al. [24] investigated the causes of entropy production rate during the condensation of a subsonic vapor flow. These investigations allow local adaptations and influences of the flow and of the geometry of a heat-transferring structure to reduce the entropy production rate due to friction, temperature gradients or fluctuation and, thus, to optimize the process or the component.

This principle of optimization is also taken up by Wenterodt [7] to numerically improve the shape of tubes in a tube-bundle as well as the shape of plates for plate heat exchangers by means of a genetic algorithm. Other approaches of this kind of optimization and evaluation of structures based on entropy production rate are presented by Jiang et al. [25] for an entropically graded heat exchanger.

As the additive manufacturing of structures allows for a huge variation in possible geometries, a fast and reliable characterization and evaluation becomes inevitable.

In this manuscript, various additively manufacturable fin structures are presented which are based on off-set strip fins for plate-fin heat exchangers. The geometric parameters, including fin height, fin spacing, fin length, and fin longitudinal displacement, are varied and their influence on the Colburn j-factor and the Fanning f-factor are investigated numerically. Subsequently, an analysis of the efficiency in terms of the irreversible entropy production rate is carried out, while the method from Bejan [8] is used due to its ease application. Additionally, the method from Bejan [8] is compared with the results of the irreversible entropy production rate calculated with the partial differential equations according to Kock [17] for a reference structure. These partial differential equations allow the calculation of the irreversible entropy production rate by heat conduction and shear stresses independently of any wall temperature definition and are, therefore, well suited for checking the method from Bejan [8].

## 2. Materials and Methods

### 2.1. Investigated Structures and Simulation Parameters

**Figure 1:**
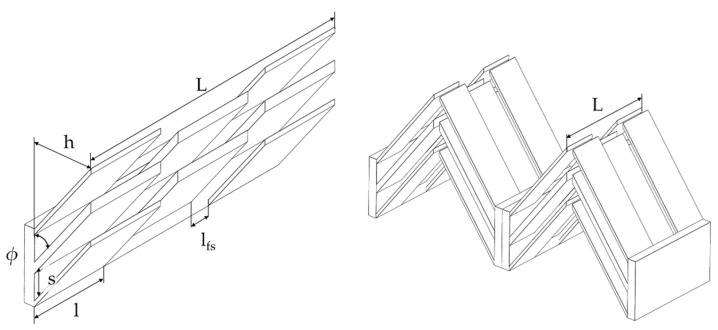
**Left**: schematic 3D model of the structures investigated with geometric parameters, and **right**: schematic 3D model of the internal fin arrangement of the investigated section.

Figure 1 shows a section of the additive off-set fin structures. The variable geometric parameters are the fin height h changed by the inclination angle ϕ, the fin spacing s, the fin length l, and the fin longitudinal displacement $l_{fs}$. 

The fin thickness and the thickness of the separation plate between the hot and cold channels remain unchanged for this investigation. The exact geometry values may not be published for reasons of intellectual property. Therefore, the ranges of the geometric parameters are given in relation to a reference structure in dimensionless form in Table 1.

**Figure 2:**
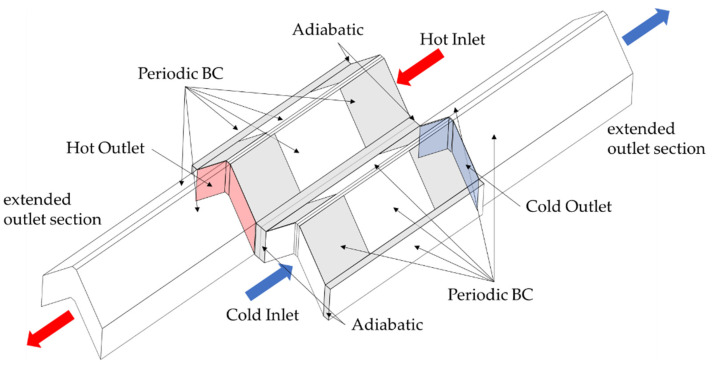
Three-dimensional model of the complete calculation domain with boundary conditions. Periodic conditions are also applied on the top grey fin and side wall areas. Evaluation of the in- and outlet temperatures and pressure drop is carried out at the coloured inlet and outlet locations.

A small section of a counterflow heat exchanger is numerically simulated, and the calculation area is shown with the corresponding boundary conditions in Figure 2. A temperature of 473 K and 353 K is specified at the hot and the cold inlet, respectively. The inlet velocity of the hot and cold sides is given as a boundary condition; on the hot side, it is varied between 3.3÷20.9 m/s, and on the cold side, it is between 2.0÷12.6 m/s, while air is used as a fluid on both sides. This corresponds to a range of Reynolds numbers (for definition, see Equation (11)) between 80 < Re < 920, depending on the exact hydraulic diameter that varies with the geometric parameters. It is important to note that the Reynolds number between the hot and cold sides only differs by 5% for all the calculations. This procedure allows the investigation of the influence of the mean fluid temperature on dissipation by shear stresses and heat conduction at the same Reynolds number.

At the outlet, a “pressure outlet” is selected as the appropriate numerical boundary condition, so that there is a free outflow against a backpressure of 101,325 Pa. Furthermore, the outlet area is extended by approx. 5 times the length of the finned area to prevent possible “reverse” flow (shortened version shown in Figure 2), which would falsify the calculation result. The front and back sides of the duct walls are assumed to be adiabatic. The material of the fins and the partition plate is a high-temperature stainless steel. Due to the small changes in the property data of the solid part in the temperature range under consideration, they are calculated at an average temperature of 413 K and stored accordingly in the calculation program ($\lambda_{s,m}=16.3\;W/\left(m\;K\right),\;c_{p,s,m}=502.5\;J/\left(\mathrm{kg}\;K\right),\;\rho_{s,m}=8030\;\mathrm{kg}/m³$. Periodic boundary conditions are selected for the remaining surfaces to allow for a realistic formation of the temperature and velocity fields. The structures are modelled using *SolidWorks 2021* [26] as well as the *DesignModeler 2021* [27]. Meshing is then performed within *Ansys Workbench 2021 R1* [27] using *Ansys Meshing 2021 R1* [27]. The meshing in the volume as well as in the inflation layers is performed with tetrahedra cells. To verify the quality of the meshing and thus the calculations, validation calculations are compared with the literature data in Section 2.4 and Section 3.1, mesh independence studies are performed in Section 3.2. The calculations are performed in *Ansys Fluent 2021 R1* [28].

### 2.2. Basic Partial Differential Equations for Mass, Momentum, Energy and Turbulence Modelling

The differential equations for mass, momentum, and energy conservation [29] are solved in the Ansys Fluent module [28]. The conservation of mass in cartesian coordinates is formulated as

(1) $$\frac{\partial \rho }{\partial t}+\nabla \cdot \left(\rho \vec{u}\right)=0$$

with $\vec{u}$ as the velocity vector in the x-, y-, and z-directions. The density ρ is calculated as an incompressible ideal gas [30], which only depends on the local temperature and not on the local pressure as the pressure drop is small. For the conservation of momentum, the following equation is applied:

(2) $$\frac{\partial \left(\rho \vec{u}\right)}{\partial t}+\nabla \cdot \left(\rho \vec{u}\vec{u}\right)=\nabla \cdot \vec{\vec{\tau }}-\nabla p+\rho \vec{g}$$

in which $\vec{\vec{\tau }}$ stands for the shear stress tensor, p for the pressure of the fluid, and $\vec{g}$ for the acceleration due to gravity. The last conservation law introduced is that of energy:

(3) $$\frac{\partial }{\partial t}\left(\rho \left(e+\frac{1}{2}{\vec{u}}^{2}\right)\right)+\nabla \cdot \left(\vec{u}\rho \left(e+\frac{1}{2}{\vec{u}}^{2}\right)\right)=\rho \left(\vec{u}\cdot \vec{g}\right)-\nabla \cdot \left(\vec{u}p\right)+\nabla \cdot \left(\vec{\vec{\tau }}\cdot \vec{u}\right)+\nabla \cdot \left(\lambda \nabla T\right)\;,$$

with *e* as the specific internal energy of the substance, λ as the thermal conductivity, and T as the thermodynamic temperature. Since the flow state (laminar/turbulent) of the fluid is not always known in advance or may change locally as the fluid flows through the structure, the flow is calculated using the Reynolds-averaged Navier–Stokes (RANS) equations to account for the effects of turbulence on velocity, temperature, and pressure. This possible local turbulence is important for the detailed calculation of the entropy production. The Navier–Stokes equations are decomposed into a time-averaged component and a fluctuation component. This results in further unknown quantities (including the Reynolds stress tensor), which must be solved and are solved here via the k-ω SST model (combination of the k-ε and k-ω models) implemented in the Ansys Fluent module. This approach can represent both far and near wall flows well via a “blending” function [31,32]. This is confirmed by the investigations of Kim et al. [33] on off-set strip fins by comparing their data to the experimentally obtained values, according to which the smallest deviations occurred with the k-ω SST model. In the k-ω SST model, the turbulent kinetic energy $k_{t}$ is calculated by the following equation:

(4) $$\frac{\partial \left(\rho \;k_{t}\right)}{\partial t}+\frac{\partial \left(\rho \;k_{t}\;u_{i}\right)}{\partial x_{i}}=\frac{\partial }{\partial x_{j}}\left[\left(\mu +\frac{\mu_{t}}{\sigma_{k}}\right)\frac{\partial k_{t}}{\partial x_{j}}\right]+G_{k}-Y_{k}+S_{k}\;,$$

and the specific dissipation rate ω via the equation

(5) $$\frac{\partial \left(\rho \;\omega \right)}{\partial t}+\frac{\partial \left(\rho \;\omega \;u_{i}\right)}{\partial x_{i}}=\frac{\partial }{\partial x_{j}}\left[\left(\mu +\frac{\mu_{t}}{\sigma_{\omega }}\right)\frac{\partial \omega }{\partial x_{j}}\right]+G_{\omega }-Y_{\omega }+S_{\omega }+\frac{2\left(1-F_{1}\right)\rho }{\omega \;\sigma_{\omega ,2}}\;\frac{\partial k_{t}}{\partial x_{j}}\;\frac{\partial \omega }{\partial x_{j}}\;.$$

The change between the k-ε model and the k-ω model takes place via the blending function F as a function of the dimensionless wall distance. The exact equations for this as well as for the turbulent viscosity and the model constants can be obtained from the *Fluent Theory Guide* [30]. The results of the calculations are the velocity, temperature, and pressure fields in the fluid, as well as the temperature field within the solid, i.e., the fin structures. The coupled solver is used as the solution algorithm; for the discretization, a 2nd-order upwind scheme is employed. The relaxation factors are not used for these calculations, and the minimum residuals are set to 1 × 10^−10^ to ensure convergence.

### 2.3. Calculation of Heat Transfer and Pressure Drop

**Figure 3:**
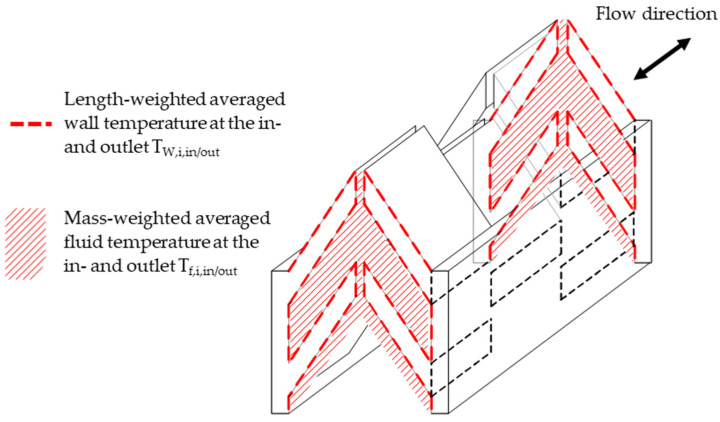
Schematic of the internal structures (hot or cold side) with locations for the wall and fluid temperatures for the determination of the heat transfer coefficient.

The heat transfer is calculated by defining the average heat transfer coefficient at a constant heat flux. In this manuscript, an average heat transfer coefficient, consisting of the heat transfer at the partition plate and around the fin structure, as well as a carefully evaluated driving temperature difference, is used for ease of application (see Figure 3). i indicates whether it is the hot (h) or the cold (c) fluid.

(6) $$\alpha_{m,i}=\frac{{\dot{Q}}_{i}}{A_{\mathrm{ht},i}\;\Delta T_{\log,i}},$$

with ${\dot{Q}}_{i}$ as the transferred heat flow rate (while ${\dot{Q}}_{c}={\dot{Q}}_{h}$, no heat flow can leave the domain because of the adiabatic front and back walls and the periodic boundary conditions).

(7) $${\dot{Q}}_{i}={\dot{m}}_{i}\;c_{p,m,i}\;{\left(T_{f,i,\mathrm{out}}-T_{f,i,\mathrm{in}}\right)}_{}$$

with $c_{p,m,i}$ as the specific isobaric heat capacity at the arithmetic mean temperature of the fluid between the inlet and the outlet, and $A_{\mathrm{ht},i}$ as heat-transferring surface (the entire internal heat-transferring area, including walls and fins). The logarithmic temperature difference is used within the local basic element as the temperature difference between the fluid and the wall to account for the constantly changing wall temperature in the direction of the flow [34], according to the following equation:

(8) $$\Delta T_{\log,i}=\frac{\left({\left(T_{W,i}-T_{f,i}\right)}_{\mathrm{in}}-{\left(T_{W,i}-T_{f,i}\right)}_{\mathrm{out}}\right)}{\ln\frac{{\left(T_{W,i}-T_{f,i}\right)}_{\mathrm{in}}}{{\left(T_{W,i}-T_{f,i}\right)}_{\mathrm{out}}}}\;\;.$$

h**.** Schematic of the internal structures (hot or cold side) with locations for the wall and fluid temperatures for the determination of the heat transfer coefficient.

In this equation, the wall and fluid temperatures right at the inlet and outlet of the heat exchanger are used (see Figure 3). For the mean fluid temperature at the inlet and outlet, the mass flow-weighted average temperature is used. For the wall temperature at the inlet and the outlet, a length-averaged temperature around the fin structures and the wall area is calculated; this applies to both the fins set at an angle and the later-introduced vertical fins. This procedure allows us to omit the separate application of the surface–fin efficiency during the evaluation, as the path-averaged fin temperature from the definition of the surface efficiency [34], Ref. [35] is already applied in the definition of the wall temperature. 

The definition of the Nusselt number is

(9) $${\mathrm{Nu}}_{i}=\alpha_{m,i}\;d_{h,i}/\lambda_{f,i}$$

with the hydraulic diameter

(10) $$d_{h,i}=4\;A_{\mathrm{cf},i}/\left(A_{\mathrm{ht},i}/L\right)$$

according to Manglik and Bergles [36], with *L* as the total length of the heat-exchanging domain. $A_{\mathrm{cf},i}$ is the cross-sectional area of the fluid at the narrowest point of the structure, i.e., the maximum Re number within the flow channel, and $A_{\mathrm{ht},i}$ is the overall heat-transferring area. The definition of the Colburn j-factor (*j*) is

(11) ji=NuiRei Pri1/3 ,

with the definition of the Reynolds number Rei=m˙iAcf,idh,i/μi. When inserting Equations (6) and (9) in Equation (11) and applying the equation for the calculation of the heat flow (7) and the hydraulic diameter (Equation (10)), the following equation for the calculation of the Colburn j-factor is obtained:

(12) $$j_{i}=\frac{{\left(T_{f,\mathrm{out}}-T_{f,\mathrm{in}}\right)}_{i}}{\Delta T_{\log,i}}\frac{A_{\mathrm{cf},i}}{A_{\mathrm{ht},i}}{\mathrm{Pr}}_{i}^{2/3}=\frac{{\left(T_{f,\mathrm{out}}-T_{f,\mathrm{in}}\right)}_{i}}{\Delta T_{\log,i}}\frac{d_{h,i}}{4\;L}{\mathrm{Pr}}_{i}^{2/3}\;.$$

The Prandtl number is calculated at the arithmetic mean temperature between the inlet and the outlet of the fluid.

In addition to the calculation of the heat transfer, the pressure drop $\Delta p_{i}$ is also of decisive importance. For this purpose, the Fanning friction factor (f) is used for the comparison of the different geometric parameters. The definition is

(13) $$f_{i}=\Delta p_{i}\frac{1}{2\;\rho_{i}}\frac{d_{h,i}}{L}\frac{1}{u_{i}^{2}}$$

with u as the mean velocity at the mean density. If the definition of the mass flow rate, $\dot{m}=A_{\mathrm{cf}}\;\rho \;u,$ is used for the calculation of u, Equation (13) can be transformed into

(14) $$f_{i}=\Delta p_{i}\;\rho_{i}\frac{d_{h,i}}{2\;L}{\left(\frac{A_{\mathrm{cf},i}}{{\dot{m}}_{i}}\right)}^{2}\;.$$

### 2.4. Validation

Before the geometry of the basic element mentioned at the beginning is calculated, the mesh settings (element size and number of inflation layers) are to be validated using the literature data. There are no validation data for the angled structures yet, so a simple rectangular off-set strip fin structure is modelled for this purpose. The geometric parameters (see Table 2) are based on the range of values of the angled geometry mentioned at the beginning in order to achieve the forming boundary layers in a comparable order of magnitude. At the same time, care is taken to ensure that the dimensionless parameters, $\beta_{f},\;\delta_{f},$ and $\gamma_{f},$ lie in the respective range of validity in which the correlations used for validation have been developed. If these can be correctly captured by the mesh of the rectangular structure, this is also to be expected to hold true for the angled structures.

**Figure 4:**
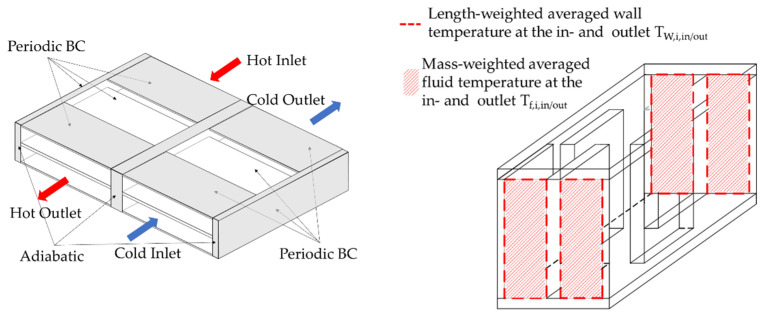
**Left**: three-dimensional model of the validation case with boundary conditions, and **right**: location of the fluid and wall temperatures for the determination of the heat transfer coefficient.

Figure 4 shows the structure for the validation and the corresponding locations for the fluid and wall temperatures to calculate the Colburn j-factor, according to Equations (6)–(12).

Equation (10) is used for the calculation of the hydraulic diameter. If the corresponding areas are calculated, Equation (10) can be rewritten for the validation case as follows:

(15) $$d_{h,\mathrm{val}}=\frac{4\;s_{f}\;h_{f}\;l_{f}}{2\;\left(s_{f}\;l_{f}+h_{f}\;l_{f}+t_{f}\;h_{f}\right)+t_{f}\;s_{f}}\;.$$

The experimentally (Manglik and Bergles [27], and Joshi and Webb [37]) and numerically based (Chennu [38,39]) equations for the j and f-factor are used for validation. Webb and Joshi [37] proposed different correlations for the Colburn j-factor and the Fanning friction factor for the laminar and turbulent regions. In this manuscript, only the two equations for the laminar region (Re < 1000) are used:

(16) jlam,JW=0.53 Re−0.5lfdh,val−0.15αf−0.14 ,

(17) flam,JW=8.12 Re−0.74lfdh,val−0.41αf−0.02 .

Likewise, Manglik et al. [36] provided the correlations for the j and f-factor, considering different geometric parameters and covering the laminar, transition, and turbulent regions. For the Colburn j-factor, the following correlation for the laminar and turbulent flow is given:

(18) jMB=0.6522 Re−0.5403 βf−0.1541 δf0.1499 γf−0.0678×1+5.269×10−5 Re1.34 βf0.504 δf0.456 γf−1.0550.1 ,

and for the f-factor, it is based on the following equation for the laminar and turbulent flow:

(19) fMB=9.6243 Re−0.7422 βf−0.1856 δf0.3053 γf−0.2659×1+7.669×10−8 Re4.429 βf0.92 δf3.767 γf0.2360.1 ,

where the accuracy of the correlations is given with ±20%.

The third comparative correlation used is that of Chennu [38,39], which was determined based on numerical simulations. Here again, a subdivision into the laminar (Re<800) and turbulent regions (1000<Re<15,000) takes place. For the Colburn j-factor and the f-factor, the following applies in the laminar region:

(20) jCh,lam=0.661 Re−0.651 βf−0.343 δf0.305 γf−0.538 ,

(21) fCh,lam=10.882 Re−0.79 βf−0.359 δf0.284 γf−0.187 .

The results of the validation process are discussed in Section 3.1.

### 2.5. Entropic Evaluation of the Structures 

In the development of heat-transferring structures or heat exchangers in general, a question usually arises which is the dissipative energy flow (as a result of heat transfer and pressure drop/shear stresses) associated with the achieved heat flow rate $\dot{Q}$. There are a lot of different comparison options for this, which Bejan [8,40], Kim et al. [41], Webb [42], and Kock [17] and Wenterodt [7], among others, have explained in detail. They also name the respective advantages and disadvantages of the individual evaluation procedure.

In this manuscript, the calculation of the irreversible entropy production rate is realized by two different methods. On the one hand, the locally resolved numerical method according to Kock [17] is applied, using partial differential equations. On the other hand, the method according to Bejan [8] is used. This method applies the same calculation parameters as for the heat transfer from the standard NTU method and the pressure drop. The method from Bejan [8] is, therefore, an approximate, but fast and easy applicable method.

#### 2.5.1. Partial Differential Equations for the Irreversible Entropy Production Rate

For the method from Kock [17], the differential equations for the calculation of the irreversible volumetric entropy production rate [7,16,17] are added in the Ansys Fluent module. Here, the equations are divided into a part associated with the fluid friction (SS) (caused by shear stresses in the fluid and on the wall) and a part related to heat conduction (HC) (in the fluid and the solid part), so ${\dot{S}}_{\mathrm{irr},\mathrm{pde}}^{\prime \prime \prime }={\dot{S}}_{\mathrm{irr},\mathrm{pde},\mathrm{HC}}^{\prime \prime \prime }+{\dot{S}}_{\mathrm{irr},\mathrm{pde},\mathrm{SS}}^{\prime \prime \prime }$ results. As with the Navier–Stokes equations, the equations for entropy calculation must be decomposed into time-averaged fractions (indicated by $\bar{□})$ and fluctuation fractions (indicated by ${□}^{\prime }$). For the time-averaged volumetric irreversible entropy production rate due to shear stress in all three spatial directions, the following applies, according to Kock [17]:

(22) $${\dot{S}}_{\mathrm{irr},\mathrm{pde},\bar{\mathrm{SS}}}^{\prime \prime \prime }=\frac{µ}{\bar{T}}\left(2\left[{\left(\frac{\partial \bar{u}}{\partial x}\right)}^{2}+{\left(\frac{\partial \bar{v}}{\partial y}\right)}^{2}+{\left(\frac{\partial \bar{w}}{\partial z}\right)}^{2}\right]+{\left(\frac{\partial \bar{u}}{\partial y}+\frac{\partial \bar{v}}{\partial x}\right)}^{2}+{\left(\frac{\partial \bar{u}}{\partial z}+\frac{\partial \bar{w}}{\partial x}\right)}^{2}+{\left(\frac{\partial \bar{v}}{\partial z}+\frac{\partial \bar{w}}{\partial y}\right)}^{2}\right)\;,$$

and the following applies for the fluctuation component when using a turbulence model [7,17,23]:

(23) $${\dot{S}}_{\mathrm{irr},\mathrm{pde},{\mathrm{SS}}^{\prime }}^{\prime \prime \prime }=\frac{\rho \;\varepsilon }{\bar{T}}=\frac{\rho }{\bar{T}}C_{µ}k_{t}\;\omega \;,$$

where *ε* stands for the isotropic dissipation rate and $C_{µ}=0.09$ for a model constant. The calculation of the total volumetric irreversible entropy production rate by shear stresses is given by ${\dot{S}}_{\mathrm{irr},\mathrm{pde},\mathrm{SS}}^{\prime \prime \prime }={\dot{S}}_{\mathrm{irr},\mathrm{pde},\bar{SS}}^{\prime \prime \prime }+{\dot{S}}_{\mathrm{irr},\mathrm{pde},{\mathrm{SS}}^{\prime }}^{\prime \prime \prime }$. 

For the irreversible entropy production rate by heat conduction in the fluid ${\dot{S}}_{\mathrm{irr},\mathrm{pde},\mathrm{HC},f}^{\prime \prime \prime }={\dot{S}}_{\mathrm{irr},\mathrm{pde},\bar{\mathrm{HC}},f}^{\prime \prime \prime }+{\dot{S}}_{\mathrm{irr},\mathrm{pde},{\mathrm{HC}}^{\prime },f}^{\prime \prime \prime }$ the following differential equation is used for the time-averaged component:

(24) $${\dot{S}}_{\mathrm{irr},\mathrm{pde},\bar{\mathrm{HC}},f}^{\prime \prime \prime }=\frac{\lambda }{{\bar{T}}^{2}}\left({\left(\frac{\partial \bar{T}}{\partial x}\right)}^{2}+{\left(\frac{\partial \bar{T}}{\partial y}\right)}^{2}+{\left(\frac{\partial \bar{T}}{\partial z}\right)}^{2}\right)\;$$

and the following short expression [7,17,23] is used for the fluctuating part:

(25) $${\dot{S}}_{\mathrm{irr},\mathrm{pde},{\mathrm{HC}}^{\prime },f}^{\prime \prime \prime }=\frac{a_{t}}{a}{\dot{S}}_{\mathrm{irr},\mathrm{pde},\bar{\mathrm{HC}},f}^{\prime \prime \prime }=\frac{\nu_{t}}{{\mathrm{Pr}}_{t}\;a}\;{\dot{S}}_{\mathrm{irr},\mathrm{pde},\bar{\mathrm{HC}},f}^{\prime \prime \prime }\;.$$

The turbulent Prandtl number is set to ${\mathrm{Pr}}_{t}=0.85$ according to [43], and the turbulent kinematic viscosity can be calculated from the effective kinematic viscosity $\nu_{t}=\nu_{\mathrm{eff}}\;–\nu$, where a is the thermal diffusivity of the fluid or the solid.

The calculation of the irreversible entropy production rate by heat conduction in the solid ${\dot{S}}_{\mathrm{irr},\mathrm{pde},\mathrm{HC},w}^{\prime \prime \prime }$ is carried out by using Equation (24), while the time-averaged values are replaced by the actual temperature in each cell since no fluctuation occurs. The overall entropy production rate by heat conduction is the sum of the part of the wall and the fluid ${\dot{S}}_{\mathrm{irr},\mathrm{pde},\mathrm{HC}}^{\prime \prime \prime }={\dot{S}}_{\mathrm{irr},\mathrm{pde},\mathrm{HC},w}^{\prime \prime \prime }+{\dot{S}}_{\mathrm{irr},\mathrm{pde},\mathrm{HC},f}^{\prime \prime \prime }$.

Finally, the total irreversible entropy production rate can be calculated via the following volume integral, as proposed by Ji et al. [23]:

(26) $${\dot{S}}_{\mathrm{irr},\mathrm{pde}}=\overset{}{\underset{V}{\int }}{\dot{S}}_{\mathrm{irr},\mathrm{pde}}^{\prime \prime \prime }dV\;.$$

#### 2.5.2. Method of Bejan [8] for Calculating the Irreversible Entropy Production Number

The method according to Bejan [8] is a lot easier to apply as it only uses the calculation variables that are used for the determination of the heat transfer and pressure drop anyway. In contrast to the method from Kock [17], Bejan [8] introduced a dimensionless entropy production number, referring the irreversible entropy production rate to the transferred entropy flow by heat. This allows a comparison of different structures and heat exchangers in terms of entropic efficiency, since a higher irreversible entropy production rate does not directly mean a more inefficient heat exchanger if the transferred heat flow is also increased. Furthermore, this entropy production number can also be interpreted as the ratio of the dissipated energy rate to the transferred heat flow rate. 

**Figure 5:**
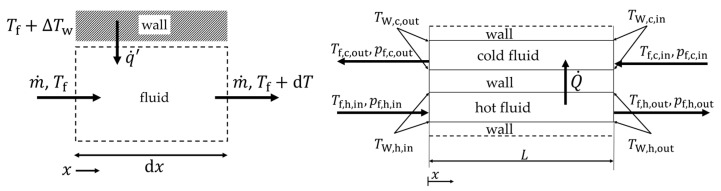
**Left**: infinitesimal fluid element for equation 27, **right**: simplified cross section of Figure 2 with the corresponding temperatures according to Figure 3. For simplification, the heat flow rate over the periodic boundary condition (dotted line) is added to the overall heat flow rate $\dot{Q}$.

For the calculation of the irreversible entropy production number, the one-dimensional differential approach according to Bejan [8] is repeated. For the case of constant heat flux (or constant length-related heat flux), the differential equation for calculating the irreversible entropy production number within a differential element *dx* of a fluid is initially as follows (see also Figure 5, left):

(27) $$N_{s}=\frac{T_{f}}{\dot{q}\prime }\frac{d{\dot{S}}_{\mathrm{irr}}}{dx}=\frac{\dot{m}}{\rho \;{\dot{q}}^{\prime }}\left(-\frac{dp}{dx}\right)+\frac{\Delta T_{w}}{T_{f}}{\left(1+\frac{\Delta T_{w}}{T_{f}}\right)}^{-1}\;.$$

Initially, Bejan [8] introduced the entropy production number only for the fluid part, so the following explanation first refers to the hot and cold fluids and is later extended to the wall part.

Equation (27) is integrated over the length *L* to obtain the entropy production number for the entire calculated section (see Figure 5, right). This leads to the following expression for the cold and hot fluids:

(28) $$N_{s,f,c}=T_{f,m,c}\frac{{\dot{S}}_{\mathrm{irr},c}}{\dot{Q}}={\left[\frac{p_{f,c,\mathrm{in}}-p_{f,c,\mathrm{out}}}{\rho_{m,c}\;c_{p,m,c}\;\left(T_{f,c,\mathrm{out}}-T_{f,c,\mathrm{in}}\right)}+\frac{\Delta T_{\log,c}}{T_{f,m,c}}{\left(1+\frac{\Delta T_{\log,c}}{T_{f,m,c}}\right)}^{-1}\right]}_{},$$

(29) $$N_{s,f,h}=T_{f,m,h}\frac{{\dot{S}}_{\mathrm{irr},h}}{\dot{Q}}={\left[\frac{p_{f,h,\mathrm{in}}-p_{f,h,\mathrm{out}}}{\rho_{m,h}\;c_{p,m,h}\;\left(T_{f,h,\mathrm{in}}-T_{f,h,\mathrm{out}}\right)}-\frac{\Delta T_{\log,h}}{T_{f,m,h}}{\left(1+\frac{\Delta T_{\log,h}}{T_{f,m,h}}\right)}^{-1}\right]}_{},$$

The first term of the equation represents the share of entropy production rate due to shear stresses (pressure drop), and the second term represents the share of entropy production rate due to heat transfer at a given temperature difference. Special attention must be paid to the temperature difference between the wall and the fluid $\Delta T_{\log,i}$ on either the hot or the cold side. In the present case, a counterflow heat exchanger configuration is investigated, and, therefore, the same logarithmic temperature difference (Equation (8)) will be used for the calculation of the heat transfer coefficient. In Section 3.2.1, the choice of this temperature difference will be reviewed. For the temperature of the fluid, again the arithmetic mean value between the inlet and the outlet is chosen as $T_{f,m,i}=\left(T_{f,i,\mathrm{in}}+T_{f,i,\mathrm{out}}\right)/2$. 

For the calculation of the irreversible entropy production number within the wall, the integral approach is used again, but the entropy production rate by shear stresses cannot occur. The wall now contains the entropy flow which enters the wall on the hot side and leaves the wall on the cold side, and possible axial heat flows leaving (or entering) the domain are not considered. The equation for the calculation of the entropy production number in the wall is then as follows:

(30) $$N_{s,W}=T_{W,m}\frac{{\dot{S}}_{\mathrm{irr},W}}{\dot{Q}}=T_{W,m}\left[{\left(\frac{1}{T_{f,m,c}+\Delta T_{\log,c}}\right)}_{}-{\left(\frac{1}{T_{f,m,h}+\Delta T_{\log,h}}\right)}_{}\right]\;,$$

with $T_{W,m}=\left[{\left(T_{f,m,c}+\Delta T_{\log,c}\right)}_{}+{\left(T_{f,m,h}+\Delta T_{\log,h}\right)}_{}\right]/2$ as the mean temperature of the wall and Equation (8) for the logarithmic wall temperature.

The total entropy production number $N_{s}$ is calculated by summing up the individual entropy production numbers to $N_{s}=N_{s,f,c}+N_{s,f,h}+N_{s,W}$. 

#### 2.5.3. Second Law Evaluation for Comparison of Both Methods

For the comparison of the calculation method from Bejan [8] and Kock [17], the irreversible entropy production rate from the second law of thermodynamics [44] is calculated by balancing around the entire calculation domain (see also Figure 2 and Figure 5, right). The only entropy flows entering and leaving are the mass-bound ones (the frontal and backward walls are taken as adiabatic), so that the following balance results:

(31) $${\dot{S}}_{\mathrm{irr},2\mathrm{nd}}={\dot{m}}_{h}\left(c_{p,m,h}\ln\frac{T_{f,h,\mathrm{out}}}{T_{f,h,\mathrm{in}}}-R\ln\frac{p_{f,h,\mathrm{out}}}{p_{f,h,\mathrm{in}}}\right)+{\dot{m}}_{c}\left(c_{p,m,c}\ln\frac{T_{f,c,\mathrm{out}}}{T_{f,c,\mathrm{in}}}-R\ln\frac{p_{f,c,\mathrm{out}}}{p_{f,c,\mathrm{in}}}\right)\;.$$

## 3. Results and Discussion

### 3.1. Validation

Table 3 shows the results of a mesh independence check for the validation case; a 3.9 mil. element mesh independence is obtained and this element number will be used for further validation calculations.

**Figure 6:**
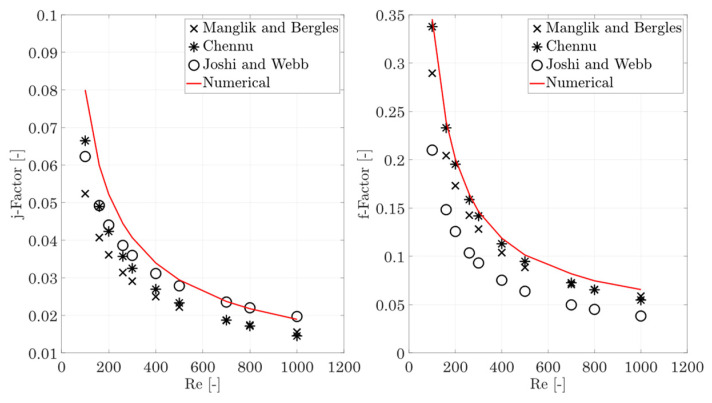
Colburn j-factor and Fanning f-factor for rectangular off-set strip fins (hot and cold sides). Comparison with values from correlations (Equations (16)–(21)) from Manglik and Bergles [36], Chennu [39], and Joshi and Webb [37].

Figure 6 shows the Colburn j-factor and the Fanning f-factor. The Colburn j-factor shows a very good agreement between the hot and cold sides. In comparison to the data from Joshi and Webb [37], the validation calculation slightly overestimates the heat transfers for small Reynolds numbers, but is still in the range of the given uncertainty of the correlation (±20%). For increasing Reynolds numbers, the deviation decreases and then shows a very good agreement.

In comparison with Manglik and Bergles [36], higher values (+35–40%) are obtained, but the mean deviation of the correlation is given as ±20% and some of the measured values show deviations up to 40%. The values of Chennu [39] are overestimated by about 15–25%. Overall, there is a systematic overprediction of the j-factor, which could be related to the chosen procedure to calculate the wall temperature, as this slightly varies from the procedure the authors used. A comparison of the f-factors shows a larger scatter for all three authors. The numerical calculations of this manuscript are rather in the upper range; for the whole Reynolds number range, a very good agreement with the values of Chennu is obtained, and for increasing Reynolds numbers, the deviation increases to approx. 15%. 

In comparison with Manglik and Bergles [36], the deviation is <15% for smaller Reynolds numbers and decreases to about 11% for high Reynolds numbers, which is also within the specified correlation accuracy. The comparison with Joshi and Webb [37] shows a significantly higher f-factor level overall, but their scattering is given within −50% to +20%. A further comparison with the validation process in Chennu [39] shows a very similar scattering of the numerically obtained j-factor and f-factor data with a larger number of different data sets. Finally, it must be considered that the off-set strip fins are comparatively complex structures, so that a larger scattering of the data for heat transfer and pressure drop must be expected, since the correlations developed may not be able to correctly represent all the effects that occur, such as manufacturing inaccuracies in the experimentally determined correlations, as also stated by Chennu [39]. Taking these aspects into account, a successful validation has been shown, so that the mesh used here can now be used as a starting point for meshing the inclined fin structures.

### 3.2. Results of the Inclined Off-Set Structures 

In the following section, the results of the inclined fin structures are presented. The two calculation methods according to Kock [17] and Bejan [8] are compared and a detailed investigation of the irreversible entropy production rate for one structure is conducted. Following this, the different geometric parameters are evaluated regarding their entropy production number, divided in the dissipation by shear stress of the cold and hot fluids and the dissipation by heat conduction as well as the overall entropy production number. For completeness, the resulting Colburn j-factor and the Fanning f-factor for the different geometric parameters are also presented. First, a mesh independence study for the inclined structures is carried out. Table 4 shows the hot-side pressure drop, the outlet temperature of the hot side, and the irreversible entropy production rate obtained by Equation (31) as well as Equation (26) for different meshes. The results show that, starting from an element number of $6.48\times 10^{6}$ elements (Mesh number 1), the change in the temperature is less than 0.02 K and the pressure drop changes by less than 0.2 Pa. The difference in the irreversible entropy production rate between Equations (26) and (31) is less than 1%. For the subsequent calculations, the mesh number 3 with $13.73\times 10^{6}$ elements is used. This ensures that the gradients for the entropy production rate are calculated correctly to overcome possible inaccuracies close to the walls, as stated by Kock [17].

#### 3.2.1. Comparison of the Calculation Methods of Bejan [8] and Kock [17]

The geometries mentioned initially are now evaluated with respect to their irreversible entropy production rate. For this purpose, the evaluation procedure is checked in advance by comparing the results for the entropy production rate according to Bejan [8] with the second law (Equation (31)) as well as the volume integral of the local entropy production rate (Equations (22)–(26)). For an easy comparison of the three different methods, the entropy production number according to Bejan [8] (Equations (28)–(30)) is converted into an irreversible entropy production rate ${\dot{S}}_{\mathrm{irr},i}=N_{s,i}\;{\dot{Q}}_{i}/T_{m,i}$ by multiplying the individual components $N_{s,f,c},\;N_{s,f,h},$ and $N_{s,W}$ (cold fluid, hot fluid, and wall) with the corresponding heat flow rate and individual mean temperatures $T_{f,m,c},\;T_{f,m,h},$ and $T_{W,m}$. Afterwards, the components are summed up to ${\dot{S}}_{\mathrm{irr},\mathrm{Bejan}}={\dot{S}}_{\mathrm{irr},f,c}+{\dot{S}}_{\mathrm{irr},f,h}+{\dot{S}}_{\mathrm{irr},W}$. 

**Figure 7:**
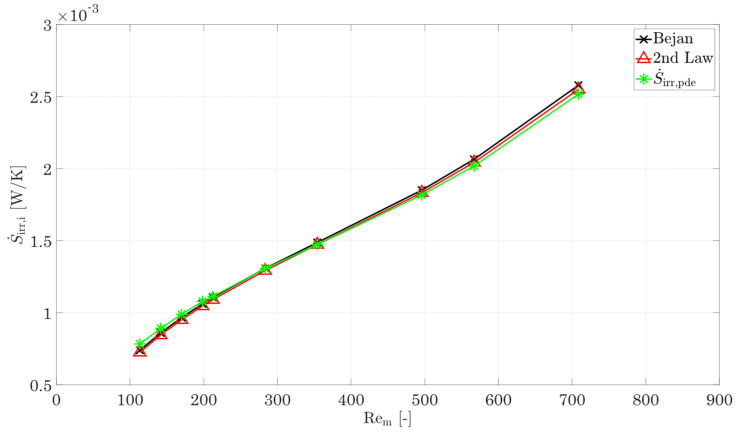
Comparison of the irreversible entropy production rate for the entire domain using the calculation method of Bejan [8], the 2nd law of thermodynamics [44], and differential equation [7,17].

Figure 7 shows the irreversible entropy production rate calculated using the integral approach (Equation (31), Bejan’s approach [8] (converted Equations (28)–(30)), and the local approach by Kock [17] (Equations (22)–(26)) depending on an arithmetic-mean Reynolds number for the hot and cold sides. The deviation between the integral approach and Bejan [8] is less than 1% over the entire flow range for the reference structure. The deviation between the local approach by Kock [17] is less than 3% on average. For very small Reynolds numbers, a deviation of 8% is calculated, and for high flow velocities, it is about 2.5%, which indicates possible residual inaccuracies of the mesh, since the calculation of the irreversible entropy production rate is performed via the square of the gradients and possible errors become more obvious [17]. Since the deviations for both variants are to be regarded as small over a wide range, both the method of Bejan [8] and the method according to Kock/Wenterodt [11], Ref. [6] are suitable for the calculation of the irreversible entropy production rate in complex structures. In the next comparison, a check of the dissipation by heat conduction and by shear stresses using the method of Bejan [8] is carried out. This comparison also reveals if the chosen logarithmic temperature difference is a suitable way to calculate the irreversible entropy production rate by heat conduction. For this purpose, the partial differential equations of Kock [17] are used, since these allow a separate calculation of the irreversible entropy production rate by heat conduction in the fluid and the wall. 

The entropy production number of the fluid ($N_{s,f,h}$ and $N_{s,f,c}$) is, therefore, separated in the part of dissipation by shear stresses $N_{s,\mathrm{SS}}$ (first term of Equations (28) and (29)) and by heat conduction $N_{s,\mathrm{HC},f}$ (second term of Equations (28) and (29)) for the hot and cold fluids. These components are then converted into the irreversible entropy production rate (as in the previous comparison) for an easy comparison with the results using Kock’s method [17].

**Figure 8:**
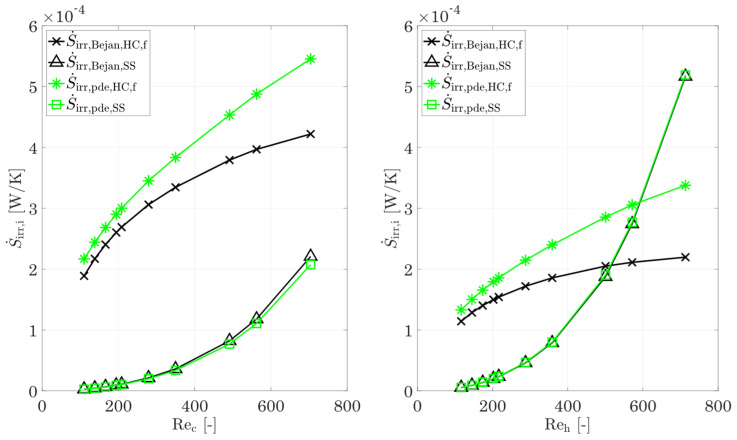
Comparison of the entropy production rate by heat conduction and shear stresses in the cold (**left**) and hot (**right**) fluids, using the method of Bejan [8] and the differential equations [17].

Figure 8 shows the irreversible entropy production rate due to heat conduction and shear stresses, according to Bejan’s method [8] as well as according to the differential equations from Kock [17] for the hot and cold fluids. An increasing deviation can be seen, in particular for the heat conduction. For small Reynolds numbers, a deviation of only 14% is shown on both the cold and the hot sides. This increases to 29% with increasing Reynolds number. For the hot side, the picture is similar: for small Reynolds numbers, the deviation is 16% and increases to 54% with increasing Reynolds number. In the same way, the irreversible entropy production rate in the fluid differs between these two methods, and the entropy production rate within the walls also shows deviations.

The discrepancies between the method of Kock [17] and the method of Bejan [8] are primarily related to the underlying choice of the temperature at which the heat flux is transported. In the local approach by Kock [17], the irreversible entropy production rate is calculated in each infinitesimal section and, thus, also underlies the temperature gradients prevailing there. In contrast, in the method proposed by Bejan [8] and also other integral methods, the entropy production rate due to heat conduction is related to a certain mean temperature definition, for example, the wall temperature [8] or the fluid mean temperature [17,44]. For complex structures, the definition of a simply defined wall temperature (such as the proposed logarithmic temperature difference in combination with the mean fluid temperature) is no longer accurate. This leads to the discrepancy between the calculated portions of entropy production rate due to heat conduction in the fluid or the wall and the true portions of entropy production rate.

In summary, the method of Bejan [8] allows an exact calculation of the entropy production rate by heat conduction for the whole heat-exchanging section (containing the hot fluid, the wall, and the cold fluid), but there is no exact subdivision on the single components due to the choice of the logarithmic temperature difference for the calculation of the wall temperature. On the other hand, Bejan’s method [8] allows an accurate calculation of dissipation due to shear stresses (see Figure 8). The deviations are less than 6% for the highest Reynolds numbers on the cold side. For smaller Re numbers, the deviations decrease to less than 1%. For the hot side, the agreement between both methods is better. For the complete Re number range, the deviations are less than 1%. If these facts are taken into account, the method of Bejan [8] allows a quick and easy classification of heat-transferring structures or complete heat exchangers with respect to the entropy production number by heat conduction in the whole component as well as the one by friction in the hot and cold fluids.

Kock’s method [17], on the other hand, allows a detailed analysis of irreversible entropy production rate, which will now be carried out on the basis of the inclined reference structure.

#### 3.2.2. Detailed Consideration of the Irreversible Entropy Production Rate on the Basis of the Inclined Reference Structure

**Figure 9:**
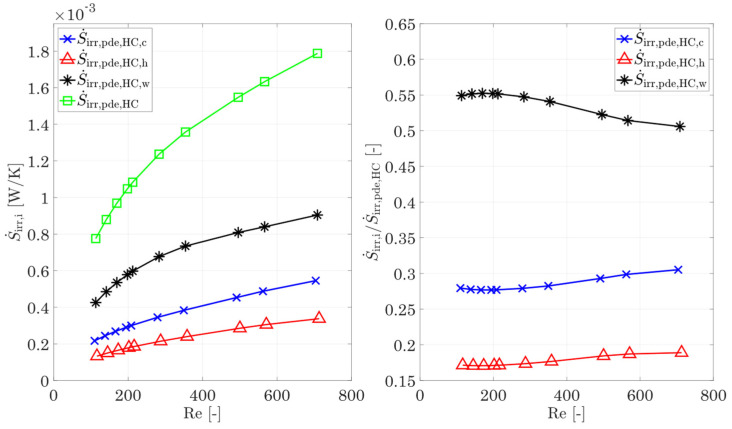
**Left:** Entropy production rate by heat conduction in the hot/cold fluid, the wall/fins, and overall entropy production rate by heat conduction, and **right**: relative proportions of the entropy generation by heat conduction of the hot/cold fluid and the wall. For the total entropy production rate and the entropy production rate in the wall and fins, the mean Reynolds number of the hot and cold sides is used.

In Figure 9, the entropy production rates for heat conduction in the fluid and the wall and the sum of the three components ${\dot{S}}_{\mathrm{irr},\mathrm{HC},\mathrm{tot}}={\dot{S}}_{\mathrm{irr},\mathrm{HC},h}+{\dot{S}}_{\mathrm{irr},\mathrm{HC},w}+{\dot{S}}_{\mathrm{irr},\mathrm{HC},c}$ for different Reynolds numbers are shown. For the representation of the course of the wall and the sum of the three partial quantities, an arithmetic average Reynolds number from the hot and cold sides is used. The plot shows that the irreversible entropy production rate in the reference structure due to heat conduction within the wall and the fin structures for the entire Reynolds number range studied are more than 50%, while the hot and cold fluids account for an average of 17.7% and 28.5%, respectively. Thus, heat conduction within the solid is the main contributor to the entropy production rate due to the temperature gradients, while the temperature gradients in the fluid due to convection and conduction have a smaller influence. Furthermore, with increasing temperature, the irreversible entropy production rate due to heat conduction in the fluid decreases as expected. However, a closer look at the *relative* contributions to the total entropy production rate by heat conduction (Figure 9, right) shows that the entropy production rate within the wall decreases with increasing Reynolds number, while the contributions from the hot and cold fluids increase. 

**Figure 10:**
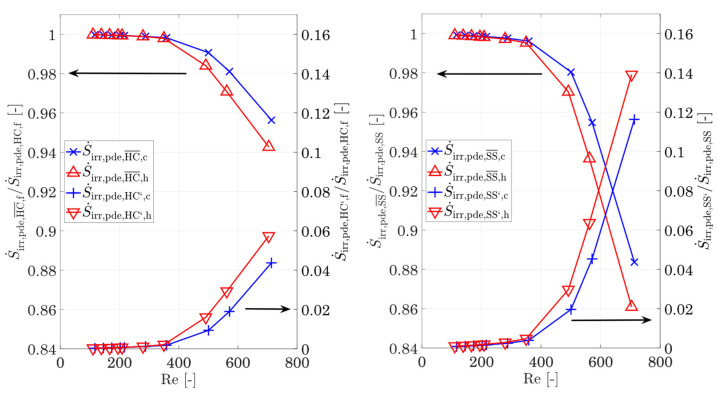
Relative proportions of the molecular and fluctuating irreversible entropy production rate by heat conduction (**left**) and shear stresses (**right**) for the hot and cold fluids.

**Figure 11:**
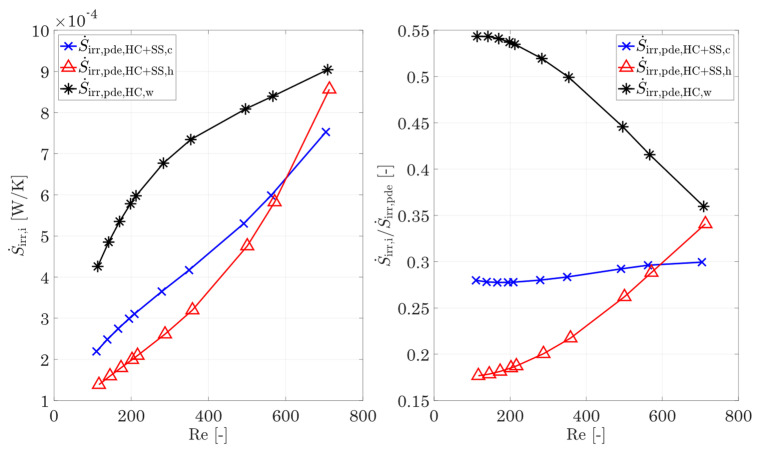
**Left:** Entropy production rate by heat conduction and shear stresses in the fluid and the wall, and **right**: relative share of the fluid and wall entropy production rates compared to the overall entropy production rate. For the entropy production rate in the wall, the mean Reynolds number of the hot and cold sides is used.

For small Reynolds numbers below 400, this increase in the fluid proportion is mainly determined by the increasing molecular heat conduction (Equation (24)). In turn, the fractions of fluctuation (Equation (25)) increase significantly and rise in the hot and cold fluids from below 0.05% at Re = 400 to 5.7% and 4.3% at Re = 713, respectively (see also Figure 10, left). With increasing Reynolds number, the degressive trend of the heat conduction of the fluid in Figure 9 is likely to turn into a progressive one, similar to the entropy production by shear stresses in Figure 8. This is also supported by the trend of the fluctuating proportions of the shear stresses in Figure 10, right. The fluctuating parts of the shear stresses are gaining significant importance for the entropy production rate and are exceeding the ones by heat conduction by a factor of 2.3 for the hot side and 2.6 for the cold side. Nevertheless, the wall remains the decisive driver for entropy production rate by heat conduction, which is why the focus should be placed on the fins, in particular when optimizing structures in order to reduce the temperature gradients. The separation of the entropy production rate by heat conduction in the wall and the fluid now also allows a separation to be made on the total entropy production rate caused by the fluid and the wall. For this purpose, Figure 11 shows the entropy production rate in the fluid and in the wall as well as their shares in the total entropy production rate (Equation (26)). With increasing Reynolds number, the influence of shear stresses on the entropy production rate in the fluid increases strongly, as shown in Figure 8, which is additionally favoured by a higher mean temperature. This finally leads to the fact that from Re = 582 onwards, the entropy production rate in the hot fluid exceeds that of the cold fluid, and for Re = 713, it almost reaches the level of the wall. It follows that, for high Reynolds numbers, the fraction of entropy production rate in the hot fluid also occupies a large fraction, while the fraction of the cold fluid increases only slightly. The entropy production rate by shear stresses dominates, despite the same Reynolds number and the total entropy production rate for increasing temperatures. It follows that, especially in high-temperature applications, very good attention must be paid to the adaptations and optimizations of the structures in terms of flow guidance and structural shaping.

**Figure 12:**
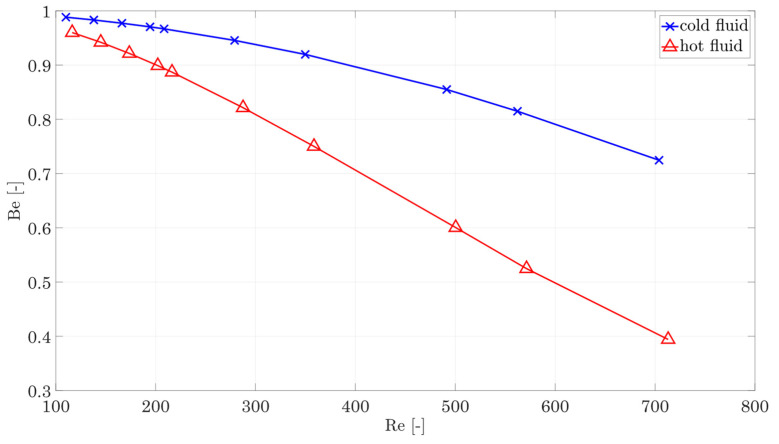
Bejan number for the hot and cold fluids.

The preceding analysis now allows the calculation of the Bejan number as a characteristic number of whether the losses in the fluid are dominated by heat conduction or shear stresses. The definition of the Bejan number [17] is as follows:

(32) $$\mathrm{Be}=\frac{{\dot{S}}_{\mathrm{irr},\mathrm{HC}}}{{\dot{S}}_{\mathrm{irr},\mathrm{HC}}+{\dot{S}}_{\mathrm{irr},\mathrm{SS}}}\;,$$

where Be→0 means the entropy production is dominated by shear stress and Be→ 1 means the entropy production is dominated by heat conduction. The curve for the hot and cold fluids based on the reference structure is shown in Figure 12. The diagram shows that the hot side, in particular, is strongly dominated by entropy generation due to shear stress as the Reynolds number increases. 

The entropy production on the cold side is dominated by temperature gradients, or heat conduction, due to the lower temperature level.

**Figure 13:**
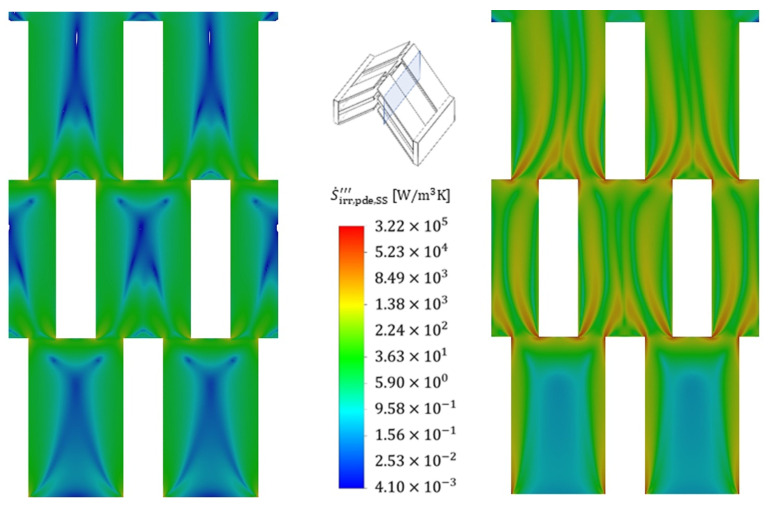
Local volumetric entropy production rate by shear stress (mean values and fluctuation) at Re = 110 (**left**) and at Re = 713 (**right**) for the cold fluid side (flow direction: bottom to top). Slicing plane for the contour plot.

To illustrate the positions within the fluid at which the entropy production rate occurs, Figure 13 shows the volumetric irreversible entropy production rate due to shear stresses (i.e., pressure drop) for two different Reynolds numbers (110 and 713) that are exemplary for the reference structure and the cold fluid. It is seen that the highest irreversible entropy production rate occurs, in particular, at the edges of the stagnation points of the structure and extends far into the wake region further downstream. The irreversible entropy production rate occurring there is 1–2 orders of magnitude higher than the general irreversible entropy production rate in the boundary layer. Furthermore, increased velocities lead to a stronger influence of the gradients and the losses due to shear stresses extend through the entire structure in a string-like manner. A comparison of the irreversible entropy production rate within the boundary layer shows an increase of about a factor of two for the increased velocity, and the entropy production rate due to shear stresses, therefore, increases strongly, as already confirmed in Figure 8. 

**Figure 14:**
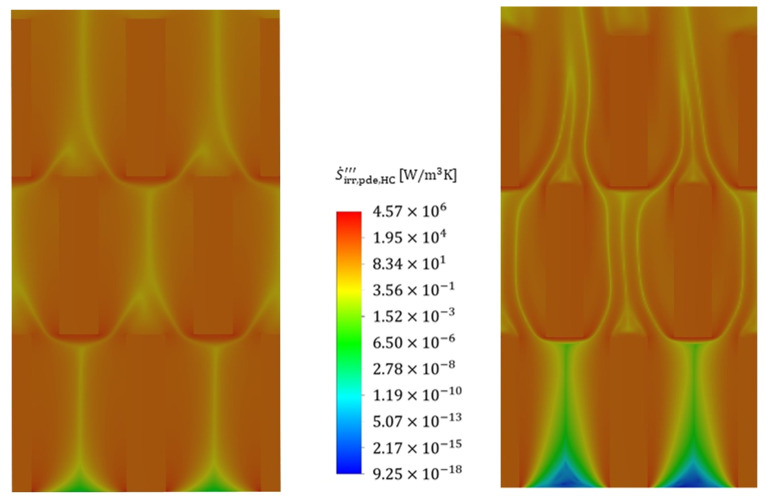
Local volumetric entropy production rate by heat conduction (mean values and fluctuating values) at Re = 110 (**left**) and at Re = 713 (**right**) for the cold fluid side.

An analysis of the thermally induced irreversible entropy production rate (Figure 14), on the other hand, shows a different picture. The dissipation due to the temperature gradients occurs on the entire front side of the fin stagnation point. After flowing around the stagnation point, the irreversible entropy production rate initially subsides somewhat as the thermal boundary layer forms, maintaining an approximately constant loss level within the thermal boundary layer, which is due to constant temperature gradients. In contrast to the fluid, the losses within the fin are homogeneously distributed, with an overall high level of losses being noticeable, confirming the findings in Figure 9.

**Figure 15:**
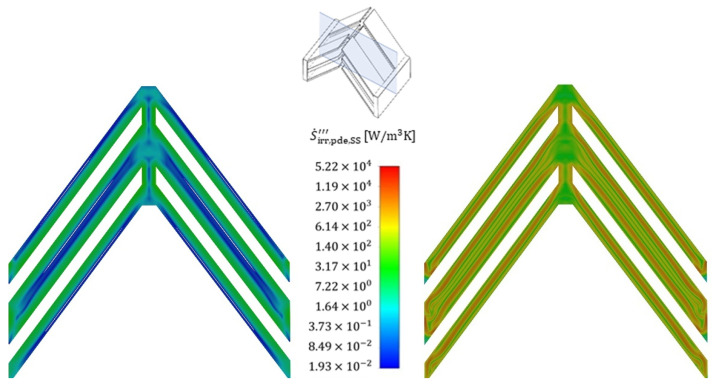
Cross-sectional view of the local volumetric entropy production rate by shear stress at Re = 110 (**left**) and at Re = 713 (**right**) for the cold fluid side. Slicing plane for the contour plot.

**Figure 16:**
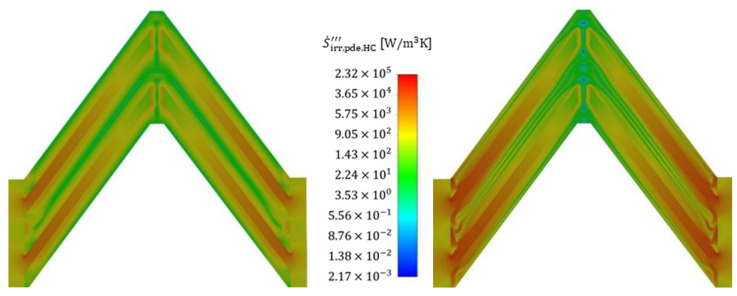
Cross-sectional view of the local volumetric entropy production rate by heat conduction at Re = 110 (**left**) and at Re = 713 (**right**) for the cold fluid side.

An increase in velocity shows a corresponding increase in irreversible entropy production rate in the areas of high gradients, i.e., particularly in the stagnation points as well as in the subsequent redirection, but this increase turns out to be much smaller than in the case with dissipation by shear stress. The irreversible entropy production rate within the fins increases only moderately, which becomes clear from the colour scaling, and this is further confirmed by Figure 9. Figure 15 and Figure 16 show the volumetric entropy production rate by shear stress and heat conduction in the cross section of the fin structures normal to the flow direction. In the case of dissipation by shear stresses, the areas of large losses with increasing flow velocity no longer occur directly at the wall, but at a short distance in front of the wall, where the turbulent fluctuation has the highest values. The smallest irreversible entropy production rate reaches the centre of the structure, where the smallest gradients are found. For the dissipation due to heat conduction, the losses within the fins vary to a considerable extent with the flow velocity, since due to the increased heat transfer, a higher heat flow has to be conducted across the fin, increasing the temperature gradients and consequently the entropy production. Furthermore, the fin structures show a significantly higher entropy production rate compared to the fluid, confirming once again that an optimization of the fin shape is considered beneficial to reduce the irreversible entropy production rate within the structure.

The local analysis, thus, allows conclusions to be drawn as to where optimization can be beneficial from an entropic point of view. To reduce the irreversible entropy production rate by shear stresses, the narrowed cross sections between two rows of fins should be mentioned. This would reduce the acceleration of the flow and, thus, reduce the shear stresses. Furthermore, optimization of the wake region could be advantageous in order to reduce the detachment area. 

Optimization with regard to entropy production rate due to heat conduction should aim at limiting the loss areas to a narrower range, i.e., reducing the boundary layer thickness and thus increasing heat transfer, for example, by thickening the downstream fin structure to match the entropy production rate, which would also increase the cross section available for heat conduction and, thus, reduce the temperature gradient along the fin height. Furthermore, an adapted fin shape, such as trapezoidal fins, can increase the cross section with decreasing distance to the wall and, thus, further lower the temperature gradient at the fin base.

On the basis of the analysis carried out, it can be stated that a detailed analysis of the occurring irreversible entropy production rate as a result of shear stresses or heat conduction is possible with the method proposed by Kock [17]. However, the calculations rely on a very fine mesh to sufficiently resolve the velocity and temperature gradients and are, thus, very computationally intensive. This mesh fineness is not absolutely necessary for the determination of heat transfer and pressure drop, as already shown by Ji et al. [23]. As a result, the method of Bejan [8] may be a simple and fast possibility to calculate the dissipation due to shear stresses and overall heat conduction. 

#### 3.2.3. Irreversible Entropy Production Number and Heat-Transferring Parameters for Different Geometric Parameters

In this subsection, the different geometric parameters are now evaluated in terms of their entropy production number using the method from Bejan [8] due to its quick application. The Colburn j-factor as well as the Fanning f-factor for the different geometric parameters are also presented.

Based on the results from “Section 3.2.1”, i.e., that the choice of the logarithmic temperature difference is not a suitable solution, we suggest a slightly different procedure compared to Bejan [8]. The entropy production number for the dissipation by shear stresses of the hot and cold fluids and the dissipation by overall heat conduction should be calculated by the following equations:

(33) $$N_{s,\mathrm{SS},c}={\left[\frac{p_{f,c,\mathrm{in}}-p_{f,c,\mathrm{out}}}{\rho_{c,m}\;c_{p,m,c}\;\left(T_{f,c,\mathrm{out}}-T_{f,c,\mathrm{in}}\right)}\right]}_{}\;,$$

(34) $$N_{s,\mathrm{SS},h}={\left[\frac{p_{f,h,\mathrm{in}}-p_{f,h,\mathrm{out}}}{\rho_{h,m}\;c_{p,m,h}\;\left(T_{f,h,\mathrm{in}}-T_{f,h,\mathrm{out}}\right)}\right]}_{}\;,$$

(35) $$N_{s,\mathrm{HC}}=T_{W,m}\left[{\left(\frac{1}{T_{f,m,c}+\Delta T_{\log,c}}\right)}_{}-{\left(\frac{1}{T_{f,m,h}+\Delta T_{\log,h}}\right)}_{}\right]+{\left[\frac{\Delta T_{\log,c}}{T_{f,m,c}}{\left(1+\frac{\Delta T_{\log,c}}{T_{f,m,c}}\right)}^{-1}\right]}_{}-{\left[\frac{\Delta T_{\log,h}}{T_{f,m,h}}{\left(1+\frac{\Delta T_{\log,h}}{T_{f,m,h}}\right)}^{-1}\right]}_{}\;.$$

One advantage of this separate consideration is that the share of dissipated power due to pressure loss as well as due to the total heat conduction is directly related to the transferred heat flow and, thus, allows a classification of the structure (or even a complete heat exchanger) with respect to energetic efficiency. The total entropy production number is then again obtained by summing up the individual entropy production numbers to give $N_{s}=N_{s,\mathrm{SS},c}+N_{s,\mathrm{SS},h}+N_{s,\mathrm{HC}}$.

Fin height h*

**Figure 17:**
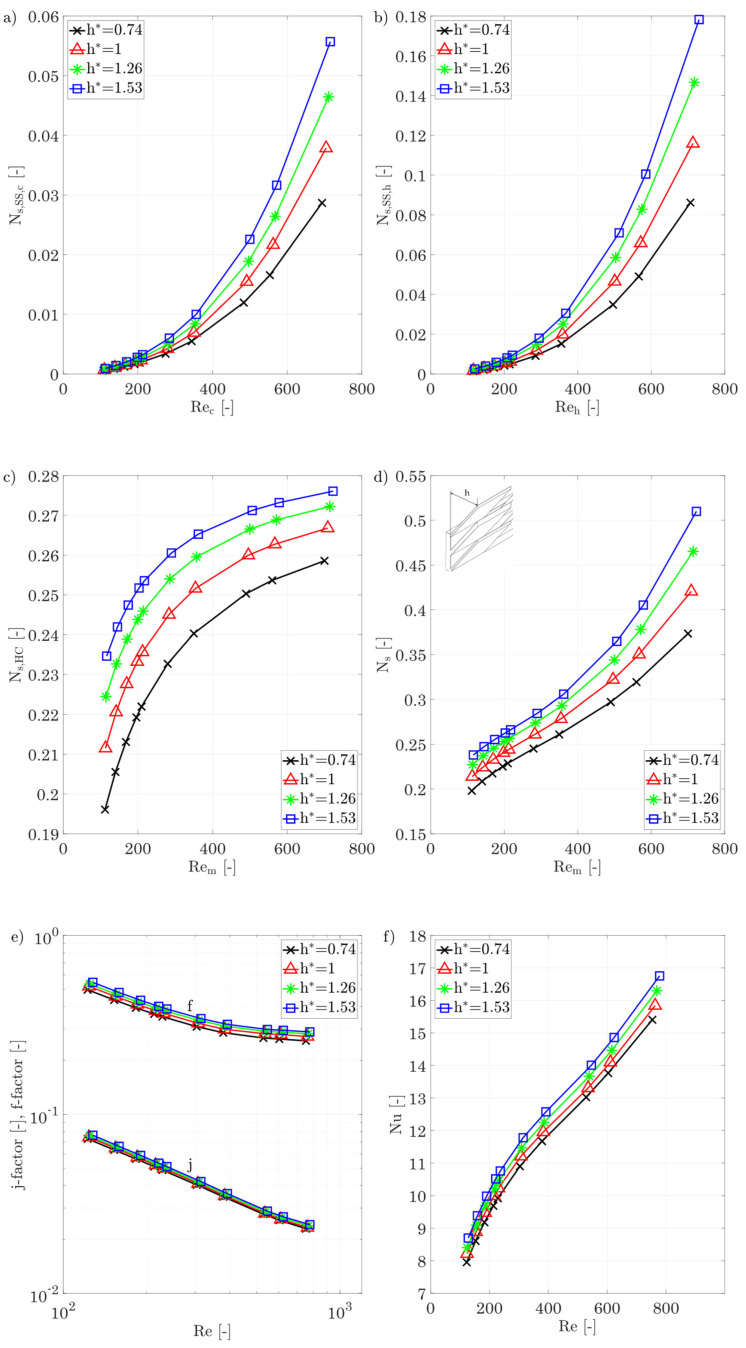
(**a**) Entropy production number due to shear stresses for the cold side, (**b**) entropy production number due to shear stresses for the hot side for different fin heights, (**c**) entropy production number due to heat conduction in the hot/cold fluid and the walls/fins, (**d**) overall entropy production number for different fin heights, and (**e**,**f**) j-factor, f-factor, and Nusselt number of the hot/cold side for different fin heights.

Figure 17 show the entropy production number for different fin heights, splitting it between hot and cold fluid dissipation by shear stresses and entropy production due to heat conduction. An average Reynolds number of the hot and cold sides is chosen for the irreversible entropy production rate due to heat conduction and the total entropy production number. In general, the entropy production rate increases with increasing Reynolds number. This is due to the strongly increasing dissipation due to shear stresses, which increases by a factor of up to 62 for the cold fluid. The entropy production rate due to shear stresses can be reduced by almost 50% as a result of a reduction in the fin height from h* = 1.53 to 0.74. For the hot side, a similar picture is shown: the entropy production number by shear stresses increases by a factor of 70 between Re = 120 and Re = 730, and a reduction in the fin height leads here to 50% lower entropy production rate due to shear stresses. The hot side shows an increased entropy production rate by a factor of about three compared to the cold side due to the increased mean temperature and the resulting increased velocity. Furthermore, it must be noted that the numerical values given in the diagrams are proportions to the losses in the entropy flux transferred, which means that for the hot side and the largest fin height investigated, almost 18% of the irreversible entropy production is due to shear stresses, while the cold side accounts for about 5.5%. An analysis of the entropy production rate due to heat conduction (which includes the hot fluid, the wall, and the cold fluid) shows a degressively increasing behavior in the Reynolds number range studied for all fin heights. For the largest fin height, the largest entropy production numbers are obtained, although the difference between the fin heights is less strongly flow dependent than is the case for shear stresses.

Based on the total entropy production number, choosing the smallest fin height at the maximum Reynolds number allows for 27.5% lower losses, while the j-factor is reduced only to a small extent. If the Reynolds number is reduced, the differences between the various fin heights are getting smaller. 

Figure 17e,f show the plots of the Colburn j-factor, the f-factor, and the Nusselt number for the different fin heights, while keeping the other geometric parameters constant; Equation (10) is again applied for the definition of the hydraulic diameter. 

The behaviour shown is also in agreement with the findings of Manglik and Bergles [36], Chennu, [39], and Joshi and Webb [37], according to which both j-factor and f-factor increase with increasing fin height. A comparison with Manglik’s [36] data also shows that the relative increase in the j- and f-factor for the inclined fins is very similar to the behaviour for straight fins. The Nusselt number only varies by a small extent with different fin heights, but indicates an increasing turbulence, since the slope of the Nusselt number increases again for Re>500.

Fin spacing s*

**Figure 18:**
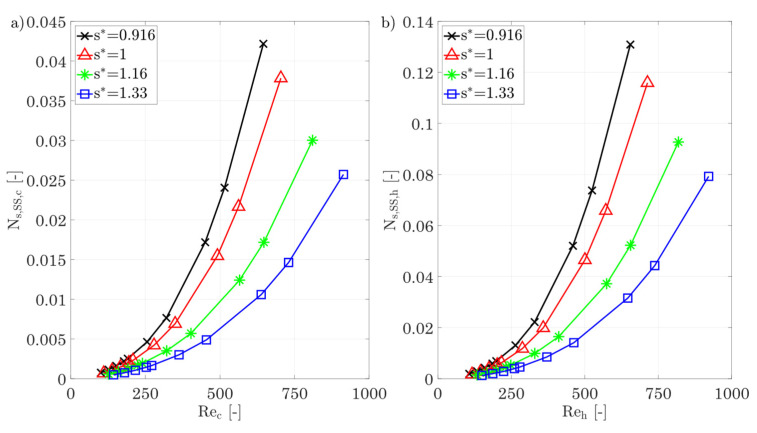

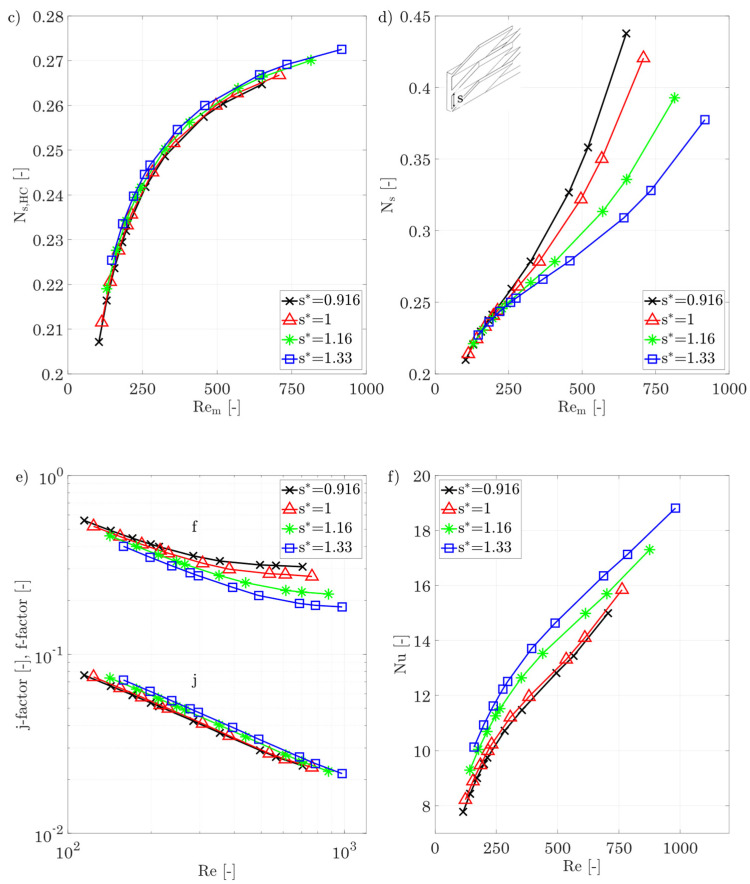
(**a**) Entropy production number due to shear stresses for the cold side. (**b**) Entropy production number due to shear stresses for the hot side for different fin spacings**.** (**c**) Entropy production number due to heat conduction in the hot/cold fluid and the walls/fins. (**d**) Overall entropy production number for different fin spacings. (**e**,**f**) j-factor, f-factor, and Nusselt number of the hot/cold side for different fin spacings.

Figure 18 shows the entropy production numbers for different fin spacings and separate them into dissipation due to shear stresses for the hot and cold sides, heat conduction in both fluids and in the wall, and the total entropy production number. The evaluation shows that for increasing fin spacing, the entropy production number due to shear stresses decreases sharply, with the hot side having higher entropy production numbers due to shear stresses, as expected, because of the higher temperature level. Using the hot and cold sides as an example, increasing the dimensionless fin spacing from 0.92 to 1.33 for a Reynolds number of 645 can reduce the entropy production rate due to shear stresses by 77% and 75%, respectively, as the velocity gradients are smaller due to the increased spacing. An analysis of the heat conduction shows that the different fin spacings have a smaller effect on the entropy production number, with the trend again being degressive as the Reynolds number increases. 

This is important because the j-factors vary more with variation in fin spacing than it is the case with fin height, but the latter shows a greater variation in entropy production number due to heat conduction. This suggests that the fraction of entropy production rate within the fluid is much smaller than within the wall. This is also plausible that varying the fin spacing does not affect the fin efficiency but varying the fin height does. A look at the total entropy production number of the heat-exchanging section shows that the increases in the entropy production number are almost entirely due to shear stresses. For an entropically favorable parameter selection, larger fin spacings should therefore be chosen, especially in areas of higher temperatures. 

Figure 18e,f show the j- and f-factor and the Nusselt number for different fin spacings as a function of the Reynolds number. In contrast to the fin height, there is a stronger dependence on the fin spacing for both the j-factor and the f-factor. The j-factor increases by up to 16% with increasing fin spacing. The f-factor shows a much stronger dependence and decreases from 0.32 to 0.2 at a Reynolds number of about 700. A comparison with the correlations of Chennu [39] shows an identical qualitative behaviour: with increasing fin spacing, the j-factor increases and the f-factor decreases. The relative change in the j-factor is of the same order of magnitude as that of Chennu’s calculation [39], but the change in the f-factor is much more pronounced.

The strong decrease in the f-factor and the simultaneous increase in the j-factor can be explained by the inclination angle of the fins. This angle creates a “gusset” in the lower region of the base of the fins, where there is a higher flow resistance due to the surrounding walls. This leads to a deflection of the flow further down into the center of the channel, with the consequence that the thermal boundary layer in the fin root area increases and, thus, the j-factor decreases, while, at the same time, the increased flow velocity in the center causes an increase in the pressure drop and consequently a higher f-factor. If the fin spacing is now increased, the flow cross section increases and reduces the f-factor. Furthermore, this also leads to a reduction in the flow resistance in the lower region of the fin root, which reduces the velocity peaks in the center and further lowers the f-factor, as well as an increase in the average flow velocity at the fin root, which reduces the thermal boundary layer, which consequently increases the j-factor. A closer look at the Nusselt number shows that, for small fin spacings, there is an increasing slope of the Nu number from Re > 500 onwards, which indicates increasing turbulence, similar to the different fin heights.

Fin length l*

**Figure 19:**
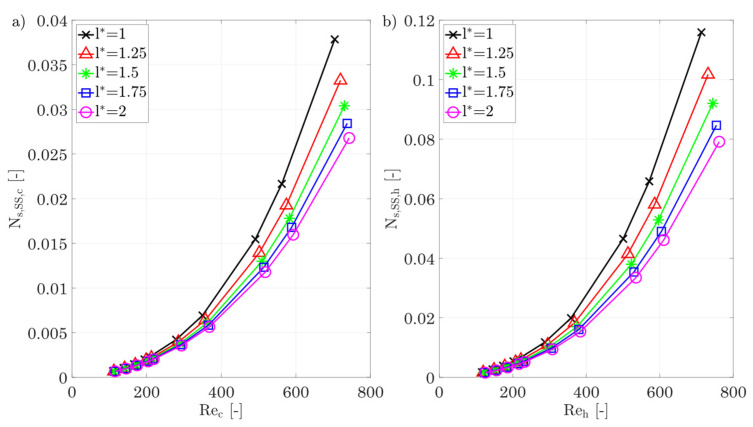

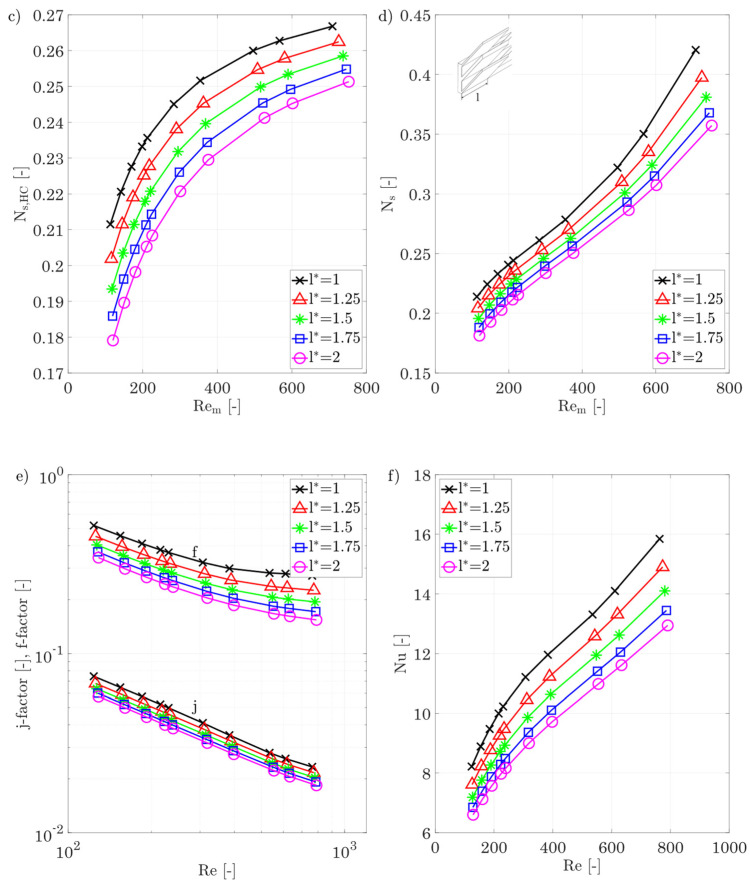
(**a**) Entropy production number due to shear stresses for the cold side. (**b**) Entropy production number due to shear stresses for the hot side for different fin lengths. (**c**) Entropy production number due to heat conduction in the hot/cold fluid and the walls/fins. (**d**) Overall entropy production number for different fin lengths. (**e**,**f**) j-factor, f-factor, and Nu number of the hot/cold side for different fin lengths.

Figure 19 shows the entropy production number as a result of different fin lengths. Basically, the entropy production decreases with increasing fin length, and this applies to shear stresses in the hot and cold fluids as well as to heat conduction. This can be explained by the decreasing temperature and velocity gradients in the fluid as well as in the wall, see also Equations (22) and (24). As the fin length increases, the fin cross section is increased, which increases the fin efficiency and, thus, reduces the entropy production rate within the fin structures. The total entropy production number shows the lowest scatter to date for varying fin lengths. Therefore, for minimum entropy production rate, a longer fin is recommended.

The j-factor, the f-factor, and the Nusselt number for different fin lengths are shown in Figure 19e,f. As expected, the Colburn j-factor and f-factor decrease with increasing fin length. This is consistent with the results of both Chennu [39], Manglik and Bergles [36], and Joshi and Webb [37], as the same effects occur. As the fin length increases, the thermal boundary layer increases and reduces the heat transfer, causing the j-factor to decrease. This is also shown by the Nusselt number, whereby doubling the fin length by 50% decreases the Nusselt number by 19%. At the same time, the hydrodynamic boundary layer also grows with increasing fin length, so that the velocity gradients are reduced, which is shown by a reduced f-factor. 

Longitudinal fin displacement l_fs_*

**Figure 20:**
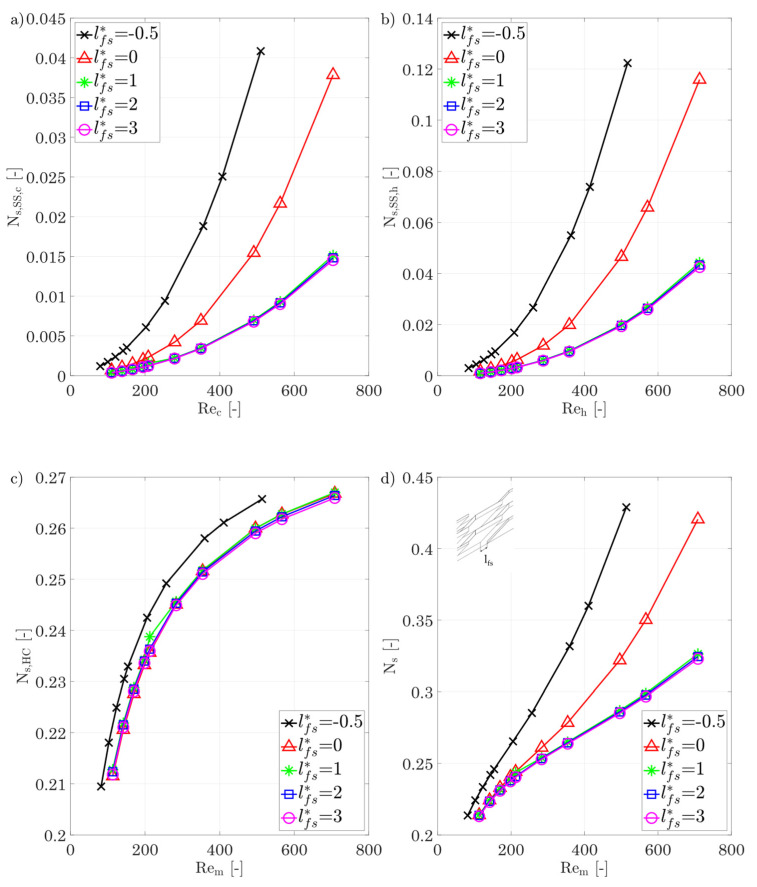

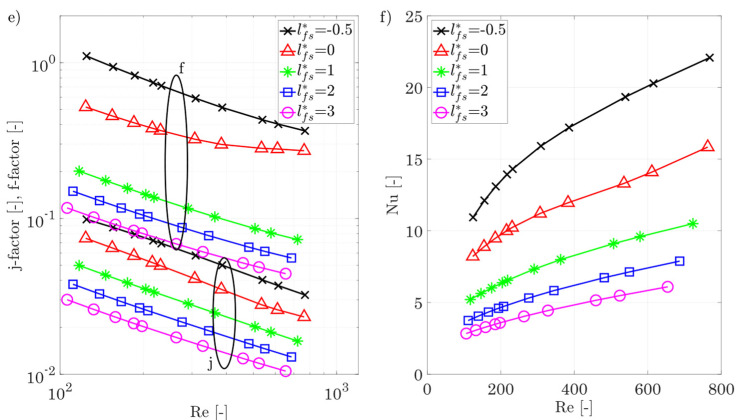
(**a**) Entropy production number due to shear stresses for the cold side. (**b**) Entropy production number due to shear stresses for the hot side for different longitudinal fin displacement. (**c**) Entropy production number due to heat conduction in the hot/cold fluid and the walls/fins. (**d**) Overall entropy production number for different longitudinal fin displacement. (**e**,**f**) j-factor, f-factor, and Nusselt number of the hot/cold side for different longitudinal fin displacement.

At last, the longitudinal fin displacement is considered. The variation in this parameter is seldom studied as this is generally associated with increased manufacturing effort using conventional methods. Additive manufacturing allows this parameter to be considered easily, which is why it is included in this investigation. In Figure 20a–d, the entropy production numbers are shown and, in general, a negative longitudinal displacement leads to a strong increase in the overall entropy production number. In the fluid, this can be explained by the strong increase in the pressure drop, or by the constantly high velocity gradient due to the thin boundary layers, which is shown by high irreversible entropy production rates due to shear stresses. It is interesting to note that a positive longitudinal fin displacement between $l_{fs}^{*}=1÷3$ shows almost no change in the entropy production number due to shear stresses. 

This means that dissipated energy due to shear stresses is reduced at the same amount as the heat flow rate, so an increase in longitudinal fin displacement does not lead to a more efficient heat exchange in terms of shear stresses. The entropy production number due to heat conduction shows, as seen already with the variation in the fin spacing, only a small dependence on the fin longitudinal displacement. This picture is also seen for the total entropy production rate: a positive longitudinal fin displacement of more than one shows almost no further reduction in the entropy production number, which, in connection with the development of the j-factor, is thus of no benefit. 

Figure 20e,f show that the j-factor and the f-factor are influenced strongly by the fin displacement. This parameter offers the greatest adaptability to heat transfer and pressure drop to reach the desired conditions to date. This can be explained by the fact that the boundary layers forming are constantly broken up and reformed, which leads to thin thermal boundary layers and high velocity gradients, resulting in high j-factors and f-factors. Further positive longitudinal displacement then shows a further reduction in the j-factor due to the increasing regions of thick boundary layers between the fins, while the relative reduction in the f-factor becomes progressively smaller. The Nusselt number can be increased by almost a factor of four when decreasing the longitudinal gap between two fin rows from 3 to −0.5.

#### 3.2.4. Interim Conclusion of the Structural Evaluation

The detailed investigation reveals that, in the laminar region, the walls and the fins are the main contributors to the entropy production rate within, while the fluid only accounts for a small amount. This might change in the turbulent region, when fluctuating components become more important. The entropy production rate due to shear stresses is highly depending on the Reynolds number and on the temperature level. Especially for higher Reynolds numbers and temperatures, the shear stresses are the main cause for the dissipation of energy and dominate the overall entropy production rate.

Based on the evaluations performed on the entropy production number, it can be conducted that, for an entropically and, thus, overall efficient heat exchanger, small fin heights, large fin spacings, long fins, and a positive longitudinal fin displacement should be selected. Furthermore, the heat exchanger should be operated at lower Reynolds numbers to keep the entropy production low.

## 4. Conclusions

In this paper, additively manufacturable fin structures of the off-set strip type were developed and a parameter study on their performance was conducted. The fin height, fin spacing, fin length, and fin longitudinal displacement were varied, and the Reynolds number were varied between 80 and 920. From these numerical calculations, the Colburn j-factors, the Fanning friction factors, and the Nusselt numbers were determined. An evaluation of the structures with respect to their irreversible entropy production rate was performed, modifying the method of Bejan [8] and comparing this method with the local entropy production rate according to Kock [17].

Comparisons of the Nusselt number and the f-factor of different fin parameters showed that variation in fin longitudinal displacement has by far the largest effect. When increasing the longitudinal displacement from −0.5 to 3, the Nu number decreases from 20 to 5 at Re = 600 and the f-factor reduces from 0.4 to 0.05. Variations in the fin length and fin spacing have less impact on the Nusselt number. Variation in the fin height has the smallest impact on the heat transfer and pressure drop, and the Nusselt number only changes by around one for the complete Re range. The f-factor changes less than 10% when varying the fin height. 

The integral method of Bejan shows deviations of less than 6% in the calculation of the irreversible entropy production rate due to pressure loss, or shear stresses, in the hot and cold fluids compared to the local method of Kock. By means of this simple method, the entropy production due to shear stresses can, thus, be calculated in a straightforward manner. 

The calculation of the irreversible entropy production by heat conduction within the fluid showed deviations between the two methods between 14 and 50%, depending on the Reynolds number, whereby for small Reynolds numbers, a better agreement is achieved. The comparison of the total irreversible entropy production caused by heat conduction shows deviations of less than 2%. The method of Bejan, thus, allows a fast classification of different structures with respect to losses due to heat conduction and pressure drop.

The detailed analysis of the irreversible entropy production shows that, for small Reynolds numbers, more than 50% of the losses are caused by heat conduction within the wall. With increasing Reynolds numbers, this share decreases to 35%, while losses due to turbulence-induced entropy production increase, especially due to shear stresses, which share rises up to 14% at high Reynolds numbers. Based on these data, entropically optimal geometric parameters can be selected with respect to heat transfer and pressure drop. Low fin height, larger fin spacing, and long fins have been shown to be advantageous with respect to the entropy production number. Furthermore, for small positive longitudinal fin displacements, a considerable reduction in the irreversible entropy production rate is shown, whereas a further increase in this distance no longer has a significant positive effect. 

In the future, the loss mechanisms in the different structures should be investigated in more detail with the aid of local entropy production rate, in order to be able to make a more precise classification for the different geometric parameters with regard to the losses due to heat conduction. In addition, the tests will also be carried out at different temperature levels in order to assess this influence more precisely. An experimental investigation of some of the structures presented in this manuscript is in preparation to validate the calculations.

## Figures and Tables

**Table 1:** Geometric parameters of the investigated structures.

$h^{*}=\frac{h}{h_{ref}}$	$s^{*}=\frac{s}{s_{ref}}$	$l^{*}=\frac{l}{l_{ref}}$	$l_{fs}^{*}=\frac{l_{fs}}{l_{fs,ref}+1}$
0.74÷1.53	0.916÷1.33	1÷2	−0.5÷3

**Table 2:** Geometric parameters for the validation case (in mm).

h [mm]	$t_{p}$ [mm]	$t_{f}$ [mm]	s [mm]	l [mm]	$d_{h}$ [mm]	$\beta_{f}=\frac{s}{h}$	$\gamma_{f}=\frac{t_{f}}{s}$	$\delta_{f}=\frac{t_{f}}{l}$
8	0.5	0.2	1.2	4	1.994	0.15	0.1667	0.05

**Table 3:** Mesh independence check for the validation case for Re = 300.

Number of Elements (Mio. Elements)	$\Delta p_{hot}$ [Pa]	$T_{f,h,out}$ [K]	$\Delta p_{cold}$ [Pa]	$T_{f,c,out}$ [K]
2.2	84.9	454.86	54.56	395.11
3.0	101.9	454.89	64.45	395.06
3.9	102.5	454.9	64.52	395.05

**Table 4:** Mesh independence of the inclined structures for Re = 300.

Mesh Number	$\Delta p_{f,h}$ [Pa]	$T_{f,h,out}\;$ [K]	${\dot{S}}_{irr,2nd}\cdot 10^{3}$ [W/K]	${\dot{S}}_{irr,pde}\cdot 10^{3}$ [W/K]
1	89.15	452.84	1.132	1.101
2	89.25	452.85	1.131	1.110
3	89.55	452.83	1.108	1.117

## Data Availability

The data presented in this study are available on request from the corresponding author. The data are not publicly available due to intellectual property.

## Nomenclature

$A_{ht}$	heat transfer area, m^2^
a	thermal diffusivity, m^2^/s
$A_{fc}$	flow cross section, m^2^
$c_{p}$	specific isobaric heat capacity, J/kg K
$d_{h}$	hydraulic diameter, m
e	specific internal energy, J/kg
*F*_1_	blending function in k-w SST model
f	Fanning friction factor,
g	gravitational acceleration, m/s^2^
$G_{K},\;G_{w}$	constant in k-w SST model
h	fin height, m
j	Colburn j-factor
$k_{t}$	turbulent kinetic energy, m^2^/s^3^
L,l	length (overall or fin length), m
$\dot{m}$	mass flow rate, kg/s
$N_{s}$	entropy production number
Nu	Nusselt number
p	pressure, Pa
Pr	Prandtl number
${\dot{q}}^{\prime }$	heat flow per unit length, W/m
$\dot{Q}$	heat flow, W
R	individual gas constant, J/kg K
Re	Reynolds number
s	(fin) spacing, m
$\dot{S}$	entropy flow, W/K
$S_{k},\;S_{w}$	constant in k-w SST model
t	fin thickness or time, m, s
T	Temperature, K
u, v, w	velocity in x-, y-, and z-directions, m/s
V	volume of domain (fluid, wall), m^3^
x, y, z	coordinate, m
$Y_{K},\;Y_{w}$	constant in k-w SST model
**Greek Letters**	
α	heat transfer coefficient, W/m^2^K
$\beta_{f},\;\gamma ,\;\delta$	dimensionless fin parameter
Δp	pressure drop, Pa
ε	isotropic dissipation rate, m^2^/s^3^
λ	thermal conductivity, W/mK
μ	dyn. viscosity, Pa s
$\mu_{t}\;$	turbulent dyn. viscosity, Pa s
ν	kinematic viscosity, m^2^/s
$\nu_{t}$	turbulent kinematic viscosity, m^2^/s
ρ	density, kg/m^3^
τ	shear stress tensor, kg/m s^2^
$\sigma_{w}$	constant in k-w SST model
ϕ	inclination angle, rad
ω	specific dissipation rate, 1/s
**Subscripts and Superscripts**	
a	heat transfer
c	cold
C	conduction
D	dissipation
eff	effective
f	fin, fluid
h	hot
HC	heat conduction
i	index (hot:h, cold:c, and wall: w)
in	inlet
irr	irreversible
lam	laminar
log	logarithmic
fs	(longitudinal) fin displacement
m	mean
out	outlet
p	plate
pde	partial differential equations
ref	reference structure
s	solid
SS	Shear Stress
t	turbulent
val	validation
w	wall
*	dimensionless fin parameter
‘’’	volumetric
$\bar{□}$	RANS mean values
${□}^{\prime }$	RANS fluctuating values
JW,MB,Ch	Joshi and Webb, Manglik and Bergles, and Chennu
2nd	2nd law analysis

## Abbreviations

RANS	Reynolds-averaged Navier–Stokes equations
